# Genetic ancestry, differential gene expression, and survival in pediatric B‐cell acute lymphoblastic leukemia

**DOI:** 10.1002/cam4.5266

**Published:** 2022-09-20

**Authors:** Freddy A. Barragan, Lauren J. Mills, Andrew R. Raduski, Erin L. Marcotte, Kelsey E. Grinde, Logan G. Spector, Lindsay A. Williams

**Affiliations:** ^1^ Department of Mathematics, Statistics, and Computer Science Macalester College St. Paul Minnesota USA; ^2^ Division of Epidemiology and Clinical Research, Department of Pediatrics University of Minnesota Minneapolis Minnesota USA; ^3^ Masonic Cancer Center University of Minnesota Minneapolis Minnesota USA

**Keywords:** acute lymphoblastic leukemia, genetic ancestry, survival disparities

## Abstract

**Background:**

Black children have lower incidence yet worse survival than White and Latinx children with B‐cell acute lymphoblastic leukemia (B‐ALL). It is unclear how reported race/ethnicity (RRE) is associated with death in B‐ALL after accounting for differentially expressed genes associated with genetic ancestry.

**Methods:**

Using Phase 1 and 2 NCI TARGET B‐ALL cases (*N* = 273; RRE‐Black = 21, RRE‐White = 162, RRE‐Latinx = 69, RRE‐Other = 9, RRE‐Unknown = 12), we estimated proportions of African (AFR), European (EUR), and Amerindian (AMR) genetic ancestry. We estimated hazard ratios (HR) and 95% confidence intervals (95% CI) between ancestry and death while adjusting for RRE and clinical measures. We identified genes associated with genetic ancestry and adjusted for them in RRE and death associations.

**Results:**

Genetic ancestry varied within RRE (RRE‐Black, AFR proportion: Mean: 78.5%, Range: 38.2%–93.6%; RRE‐White, EUR proportion: Mean: 94%, Range: 1.6%–99.9%; RRE‐Latinx, AMR proportion: Mean: 52.0%, Range: 1.2%–98.7%). We identified 10, 1, and 6 differentially expressed genes (*p*_adjusted_ <0.05) associated with AFR, AMR, and EUR ancestry proportion, respectively. We found AMR and AFR ancestry were statistically significantly associated with death (AMR each 10% HR: 1.05, 95% CI: 1.03–1.17, AFR each 10% increase HR: 1.03, 95% CI:1.01–1.19). RRE differences in the risk of death were larger in magnitude upon adjustment for genes associated with genetic ancestry for RRE‐Black, but not RRE‐Latinx children (RRE‐Black HR: 3.35, 95% CI: 1.31, 8.53; RRE‐Latinx HR: 1.47, 0.88–2.45).

**Conclusions:**

Our work highlights B‐ALL survival differences by RRE after adjusting for ancestry differentially expressed genes suggesting other factors impacting survival are important.

## Graphical abstract

Using genotype, gene expression, and survival data, we analyzed the relationship between race/ethnicity and survival in pediatric B‐cell acute lymphoblastic leukemia (B‐ALL), when accounting for genes associated with genetic ancestry. Our results suggest that genetic ancestry and its associated genes play meaningful roles in the occurrence and magnitude of racial/ethnic disparities in B‐ALL.

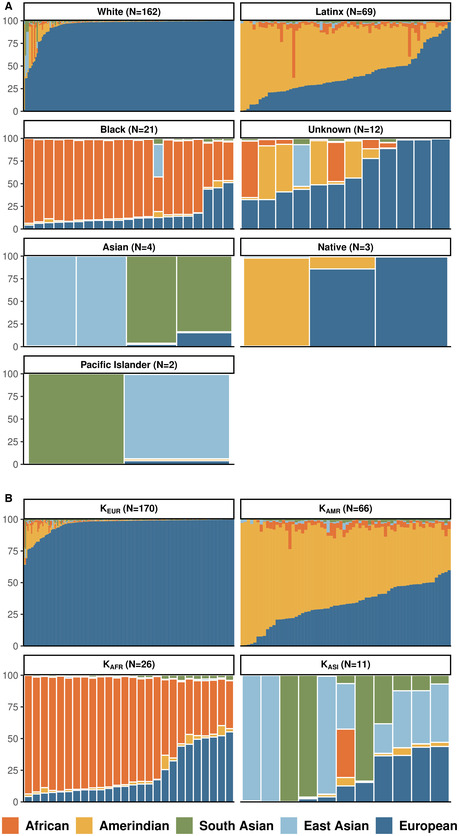

## 1 INTRODUCTION

There are differences in acute lymphoblastic leukemia (ALL) incidence where Black children have half the rate of White children^1^, ^2^, ^3^ and Latinx children have 1.2 times the rate of White children.^4^ However, Black children experience a 50% and Latinx children 20% excess mortality over Whites in recent treatment eras^5^, ^6^, ^7^ only partially due to higher non‐adherence to maintenance therapy^8^ or socioeconomic status.^9^ Latinx children have higher rates of relapse than White.^10^ The differences in clinical presentation and outcomes between Black, Latinx and White children with ALL may be partially explained by genetic ancestry.

Some work has been done for genetic ancestry and B‐ALL outcomes as there is a large and established literature discussing the discordance, methodologically and biologically, between RRE and genetic ancestry in disparities research.^11^, ^12^, ^13^, ^14^ One multiethnic genome wide association study (GWAS) determined genetic ancestry of cases^15^ and found five‐year cumulative incidence of relapse, a major cause of B‐ALL mortality, was 24% in Black children and 10% in White; however, this association was attenuated to 7% for Black children after adjustment for four variants most strongly associated with relapse.^15^ A recent report by Lee et al. (2022) of over 2300 cases of B‐ALL found that every 25% increase in African ancestry was associated with poorer event free survival even after adjusting for cytogenomic subtype and clinical factors.^16^ The evidence to date suggests that genetic ancestry is important in B‐ALL outcomes, but has failed to account for gene expression differences between ancestry groups independent of the prognostic cytogenomic subtypes. Therefore, we estimated survival differences and identified differentially expressed genes associated with African, European, and Amerindian genetic ancestry in children with B‐ALL from NCI's TARGET initiative to obtain a more complete picture of the biologic underpinnings of survival disparities in B‐ALL.

## 2 METHODS

The results herein are based on data generated by the TARGET initiative (https://ocg.cancer.gov/programs/target), phs000218 (https://portal.gdc.cancer.gov/projects).

### 2.1 Study population

Children aged 0–19 years diagnosed with microscopically confirmed, first primary cancers were identified in TARGET (data retrieved: 06/05/2019). Data from B‐ALL samples were used from Acute Lymphoblastic Leukemia Pilot [ALL P1] dbGaP accession: phs000463; Acute Lymphoblastic Leukemia Expansion [ALL P2] dbGaP accession: phs000464. We obtained germline data (Affymetrix SNP 500 K [Phase 1], Affy SNP 6.0 [Phase 2]) for genetic ancestry inference and somatic gene expression data [Affymetrix U133 Plus 2 microarrays] to infer subtype and identify differentially expressed genes from TARGET for primary diagnosis B‐ALL cases. As the data are publicly available this study was exempt from University of Minnesota Institutional Review Board review.

### 2.2 Cytogenomic subtype inference

B‐ALL cytogenomic subtype in cases with missing information (62.5%) was determined using random forests (RF) trained on subsets of ‘informative’ genes (Figure S1) selected by a RF classifier with out‐of‐bag accuracy metrics trained on gene expression data from the 10,000 most variable genes. Using the informative genes (Gini Index >95th percentile), we performed principal component decomposition of gene expression data and implemented a RF classifier with thrice repeated 10‐fold cross validation using the first 10 principal components to classify B‐ALL subtypes on cases with missing information. The proposed RF subtype classifier demonstrated good prediction metrics after multiple rounds of cross‐validation with an overall cross‐validated accuracy of 81.5%. Due to small sample sizes upon stratification by RRE/genetic ancestry, the following inferred subtypes were excluded: Hypodiploid (*N* = 11), BCR‐ABL1 (*N* = 8), iAmp 21 (*N* = 8), and TCF3‐HLF positive cases (*N* = 1). Trisomy refers to a trisomy of both chromosomes 4 and 10. Hyperdiploid was determined prior to analysis by TARGET and includes individuals with definite hyperdiploidy and either no or unknown trisomy of chromosomes 4 and 10.

### 2.3 Reported race/ethnicity (RRE)

Among cases, RRE was Asian (*N* = 4), Black (*N* = 21), Latinx (*N* = 69), Native (*N* = 3), Pacific Islander (*N* = 2), White (*N* = 162) and unknown (*N* = 12). We refer to individual RRE groups in our study by these reported categories and reiterate that genetic ancestry is distinct from RRE.

### 2.4 Ancestry inference

We derived reference panels from the 1000 Genomes Project^17^ with 504 African (AFR), 347 Amerindian (AMR), 504 East Asian (EAS), 412 European (EUR), and 489 South Asian (SAS) individuals with phased 30× autosomal whole genome sequencing data. TARGET genotype data (*N* = 286) was processed using standard quality control metrics such that SNPs included had missing rate <20%, Hardy–Weinberg equilibrium *p*‐value >0.0000001, and a minor allele frequency >1%, leaving 199,145 SNPs in Phase 1 and 329,041 SNPs in Phase 2. Haplotype phasing was performed using ShapeIt4.^18^ Local and global ancestry were inferred using RFMix (v.2.03‐r0)^19^ with window and generation parameters set to 0.2 cM and 8 generations since admixture, respectively.^20^, ^21^ Using each sample's five global ancestry proportions (South Asian, East Asian, Amerindian, African, European), we implemented a K‐means clustering algorithm to classify children into four groups of predominantly AFR (K_AFR_), AMR (K_AMR_), EUR (K_EUR_), and broadly Asian (K_ASI_) ancestry (Figure S2). All survival and gene expression analyses were restricted to RRE‐White, RRE‐Latinx, and RRE‐Black children (*N* = 264) or children whose ancestry clusters indicated predominant EUR, AMR, and AFR genetic ancestry (K_AFR_, K_AMR_, K_EUR_; *N* = 262).

### 2.5 Differential gene expression

Expression data were processed and normalized using the Affymetrix Microarray Analysis Software (MAS 5.0) on Affymetrix U133 Plus 2.0 Arrays. Prior to analysis, we removed batch effects by TARGET Phase then averaged repeat gene expression measurements by sample and probe ID. Using *limma* (v.3.48.1), we performed differential expression (DE) analyses to identify genes whose expression was statistically significantly associated (Benjamini‐Hochberg *p*‐value <0.05) with each 10% increase in AFR, AMR, and EUR ancestry proportions, when adjusting for TARGET phase and B‐ALL subtype (ETV6‐RUNX1, Hyperdiploid, MLL, TCF3‐PBX1, Trisomy [both chromsomes 4 and 10]). In addition to these continuous measures of ancestry, we also performed DE analyses between ancestry clusters (K_EUR_ vs. K_AFR_, K_EUR_ vs. K_AMR_, and K_AMR_ vs. K_AFR_).

### 2.6 Survival analysis

Kaplan Meier survival curves and Log‐Rank *p*‐values were used to identify differences in 15‐year survival between RRE groups (RRE‐White, RRE‐Latinx, RRE‐Black); ancestry clusters (K_EUR,_ K_AMR,_ K_AFR_,); EUR, AMR, and AFR ancestry proportion categories defined a priori as 0%–25%, 25%–75%, and 75%–100%; and median‐stratified categories of ancestry proportion within RRE groups (EUR within RRE‐White, AMR within RRE‐Latinx, and AFR within RRE‐Black).

Using Cox regression models, we estimated hazard ratios (HR) and 95% confidence intervals (95% CI) as the measure of association between RRE (RRE‐White referent)/K‐means ancestry clusters (K_EUR_ referent) and death. For ancestry proportion, we estimated HRs and 95% CIs between each 10% increase in EUR, AMR, and AFR ancestry and death in single ancestry models (0% as referent). To capture survival variation by increasing genetic ancestry proportions within RRE groups, we estimated HR and 95% CI between increasing ancestry proportion (EUR in RRE‐White, AFR in RRE‐Black, and AMR in RRE‐Latinx) and death.

To identify DE genes whose log_2_ mean expression values were significantly associated with death, we estimated HR and 95% CIs between mean expression and death in multiple independent Cox proportional hazard models, with additional adjustment for RRE. We then fit 3 separate Cox proportional hazard models of the relationship between RRE and death for all children, when adjusting for (1) log_2_ mean expression of all DE genes, (2) DE genes associated with survival after multiple testing adjustment (BH‐*p* < 0.05), and (3) all DE genes associated with survival (*p* < 0.05).

Unless specified, adjustment sets were the same in each of the aforementioned survival models and included: sex [male, female], B‐ALL risk group^22^ [low risk: 1–10 years of age; high risk: <1 or >10–19 years of age], WBC count [low risk: WBC >50,000/μl; high risk: WBC <50,000/μl], CNS involvement (low risk: 0 blasts in cerebrospinal fluid [CSF], high risk: >0 blasts in CSF), and inferred subtype (ETV6‐RUNX1, Hyperdiploid, MLL, TCF3‐PBX1, Trisomy [both chromosomes 4 and 10]). We found no violation of the proportional hazard assumptions in any models using standard evaluation metrics, including Schoenfield residuals (all *p* > 0.05). Statistical significance was determined using two‐sided hypothesis tests with an alpha of 0.05.

### 2.7 Software

Genetic ancestry inference was performed using Python (v.3.8.5), R (v.4.1.0), and RFMix (v.2.03‐r0). Quality control, phasing, and ancestry inference code can be found here: https://github.com/pmonnahan/AncInf. All remaining analyses and figures were generated with RStudio (v.1.4.1103).

## 3 RESULTS

Clinical characteristics are listed in Table 1 (*N* = 264). RRE‐White children (*N* = 162) were the majority (61.4%), followed by RRE‐Latinx (26.1%) and RRE‐Black (8%). The study sample was largely male, aged 0–4 years at diagnosis, and without CNS involvement. Hyperdiploid B‐ALL was the most common cytogenetic subtype (overall 40.5%; RRE‐White: 37.7%; RRE‐Latinx: 47.8%). Among RRE‐Black children, *TCF3‐PBX1* was most common (33.3%). When stratified by RRE, RRE‐Black children had the highest percentage of deaths (57.1%).

**TABLE 1:** Demographic information for RRE‐White, RRE‐Black and RRE‐Latinx children with B‐ALL from the NCI TARGET initiative Phases 1 and 2, (2004–2010)

	White	Black	Latinx	Unknown	Total
	*N* (%)	*N* (%)	*N* (%)	*N* (%)	*N* (%)
RRE					
White	162 (100)	0 (0)	0 (0)	0 (0)	162 (61.4)
Black	0 (0)	21 (100)	0 (0)	0 (0)	21 (8.0)
Latinx	0 (0)	0 (0)	69 (100)	0 (0)	69 (26.1)
Unknown	0 (0)	0 (0)	0 (0)	12 (100)	12 (4.5)
Sex					
Female	66 (40.7)	4 (19.0)	28 (40.6)	2 (16.7)	100 (37.9)
Male	96 (59.3)	17 (81.0)	41 (59.4)	10 (83.3)	164 (62.1)
Age at diagnosis					
0–4	68 (42.0)	10 (47.6)	26 (37.7)	4 (33.3)	108 (40.9)
10–14	52 (32.1)	4 (19.0)	24 (34.8)	4 (33.3)	84 (31.8)
15–19	42 (25.9)	7 (33.3)	19 (27.5)	4 (33.3)	72 (27.3)
TARGET year of diagnosis					
2004–2006	142 (87.7)	15 (71.4)	55 (79.7)	10 (83.3)	222 (84.1)
2007–2008	17 (10.5)	5 (23.8)	13 (18.8)	2 (16.7)	37 (14.0)
2009–2010	3 (1.9)	1 (4.8)	1 (1.4)	0 (0)	5 (1.9)
Molecular subtype					
ETV6‐RUNX1	22 (13.6)	5 (23.8)	8 (11.6)	1 (8.3)	36 (13.6)
Hyperdiploid	61 (37.7)	4 (19.0)	33 (47.8)	9 (75.0)	107 (40.5)
MLL	35 (21.6)	0 (0)	9 (13.0)	1 (8.3)	45 (17.0)
TCF3‐PBX1	24 (14.8)	7 (33.3)	12 (17.4)	1 (8.3)	44 (16.7)
Trisomy	20 (12.3)	5 (23.8)	7 (10.1)	0 (0)	32 (12.1)
WBC count (1000/μl)					
Mean (SD)	89.5 (154)	31.1 (37.6)	94.7 (141)	49.6 (73.1)	84.4 (142)
Median [Min, Max]	36.7 [1.00, 1150]	17.8 [2.60, 153]	39.8 [3.50, 959]	10.2 [1.30, 194]	33.5 [1.00, 1150]
CNS involvement					
0 Blasts	131 (80.9)	18 (85.7)	54 (78.3)	10 (83.3)	213 (80.7)
1–5 Blasts	21 (13.0)	2 (9.5)	9 (13.0)	1 (8.3)	33 (12.5)
>5 Blasts	10 (6.2)	1 (4.8)	6 (8.7)	1 (8.3)	18 (6.8)
Vital status					
Alive	106 (65.4)	9 (42.9)	35 (50.7)	7 (58.3)	157 (59.5)
Dead	55 (34.0)	12 (57.1)	34 (49.3)	5 (41.7)	106 (40.2)
Unknown	1 (0.6)	0 (0)	0 (0)	0 (0)	1 (0.4)

**FIGURE 1:**
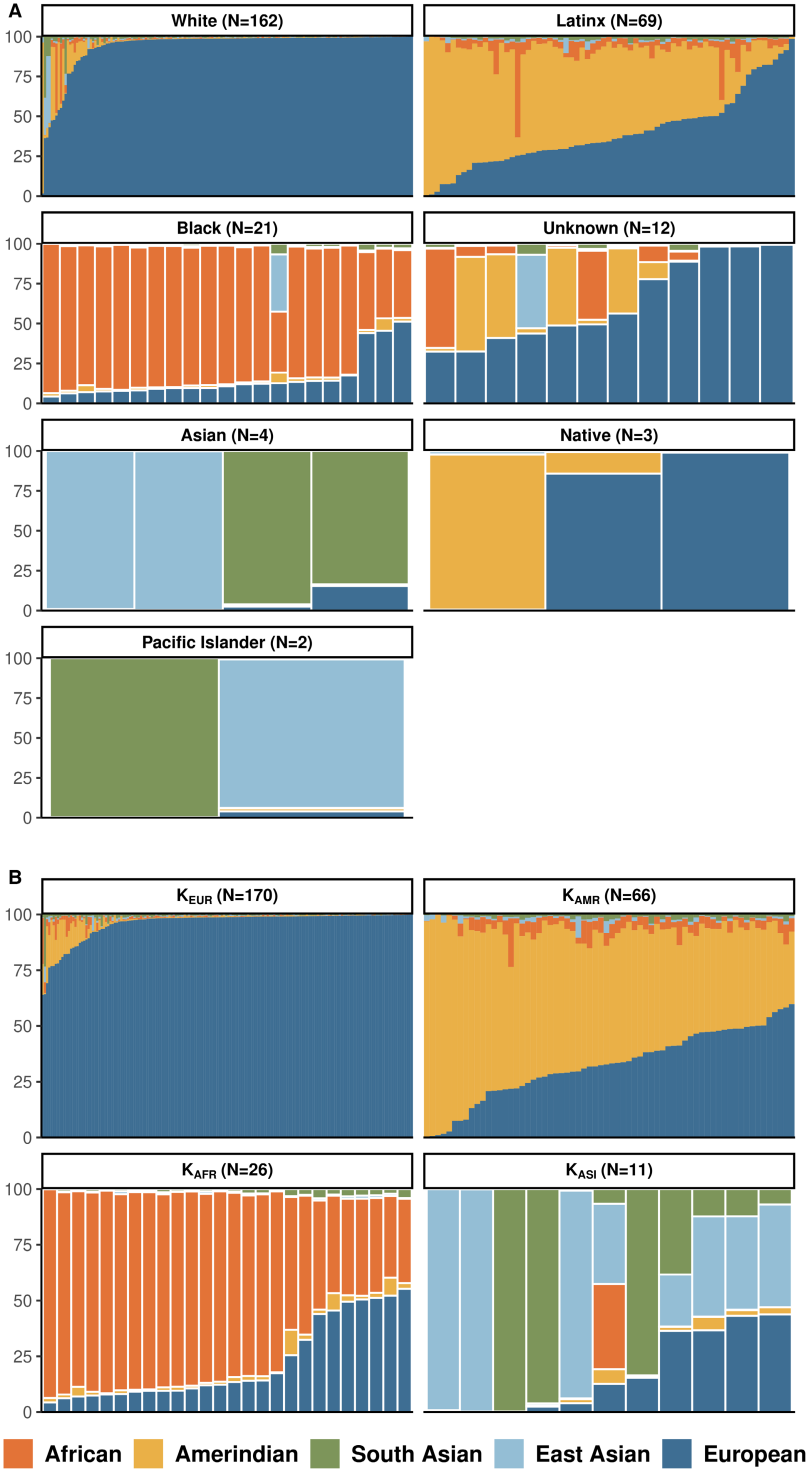
(A) Ancestry cluster for children with B‐ALL results using K‐means methods by proportion of reported race/ethnicity (RRE); (B) Genetic ancestry proportions for children with B‐ALL by reported race/ethnicity (RRE), colored by ancestral population (Orange = African, Yellow = Amerindian, Green = South Asian, Light Blue = East Asian, Dark Blue = European). NCI TARGET B‐ALL cases (2004–2010)

Global ancestry estimates revealed variation in genetic ancestry within RRE groups (Figure 1). RRE‐White children presented with the highest proportion of European (EUR) genetic ancestry (Median: 98.8%, Range: 1.7%–99.9%), yet RRE‐Latinx (Median: 34.2%, Range: 0.3%–99.5%) and RRE‐Black (Median: 34.2%, Range: 4.3%–51.2%) children presented with nontrivial proportions of EUR. Amerindian (AMR) genetic ancestry was most common among RRE‐Latinx children (Median: 55.0%, Range: 1.2%–98.7%); however, 27 of the 162 RRE‐White children had AMR ancestry >1% (RRE‐White AMR Median: 0.3%, Range: 0.0%–94.6%; RRE‐Latinx AMR Median: 55.0%, Range: 1.2%–98.7%; RRE‐Black AMR Median: 1.7%, Range: 0.4%–7.8%). High African (AFR) genetic ancestry proportions were concentrated in RRE‐Black children except for a few RRE‐Latinx and RRE‐White children (RRE‐White AFR Median: 0.2%, Range: 0.0%–43.6%, *N* = 23 AFR >1%; RRE‐Latinx AFR Median: 4.03%, Range: 0.07–59.5% *N* = 64 AFR >1%; RRE‐Black AFR Median: 86.6%, Range: 38.2%–93.6%).

K‐means clustering resulted in three clusters of predominantly AFR (*N* = 26), AMR (*N* = 66), and EUR (*N* = 170) ancestry and 1 cluster of broadly EAS and SAS ancestry (*N* = 11; Figure 1B). There was heterogeneity of RRE within ancestry clusters (Figure S3). Ten RRE‐White children (6.17%) had predominantly non‐EUR ancestry (K_AFR_ = 2; K_AMR_ = 5; K_ASI_ = 3), 13 RRE‐Latinx children (18.84%) had predominantly non‐AMR ancestry (K_AFR_ = 2; K_AMR_ = 11), and one RRE‐Black child (4.76%) was identified as having predominantly Asian ancestry (K_ASI_). Ancestry proportions within predominantly K_EUR_ children were similar to RRE‐White children, but with more homogeneity in EUR (Median: 98.80%, Range: 64.0%–99.9%), AFR (Median: 0.2%, Range: 0.0%–20.7%), and AMR (AMR Median: 0.3%, Range: 0.0%–25.5%) ancestry proportions. Likewise, ancestry proportions within K_AMR_ mirrored RRE‐Latinx children, but resulting ranges were smaller for all ancestry proportions (EUR Median: 33.0%, Range: 0.3%–59.8%; AFR Median: 4.0%, Range: 0.04%–21.8%; AMR Median: 57.7%, Range: 28.2%–98.7%). Among K_AFR_ children, there was a larger range of AFR ancestry proportions (Median: 83.7%, Range: 36.7%–93.6%) and increased EUR (Median: 12.9%, Range: 4.3%–55.3%) and AMR (Median: 1.9%, Range: 0.4%–11.4%).

**FIGURE 2:**
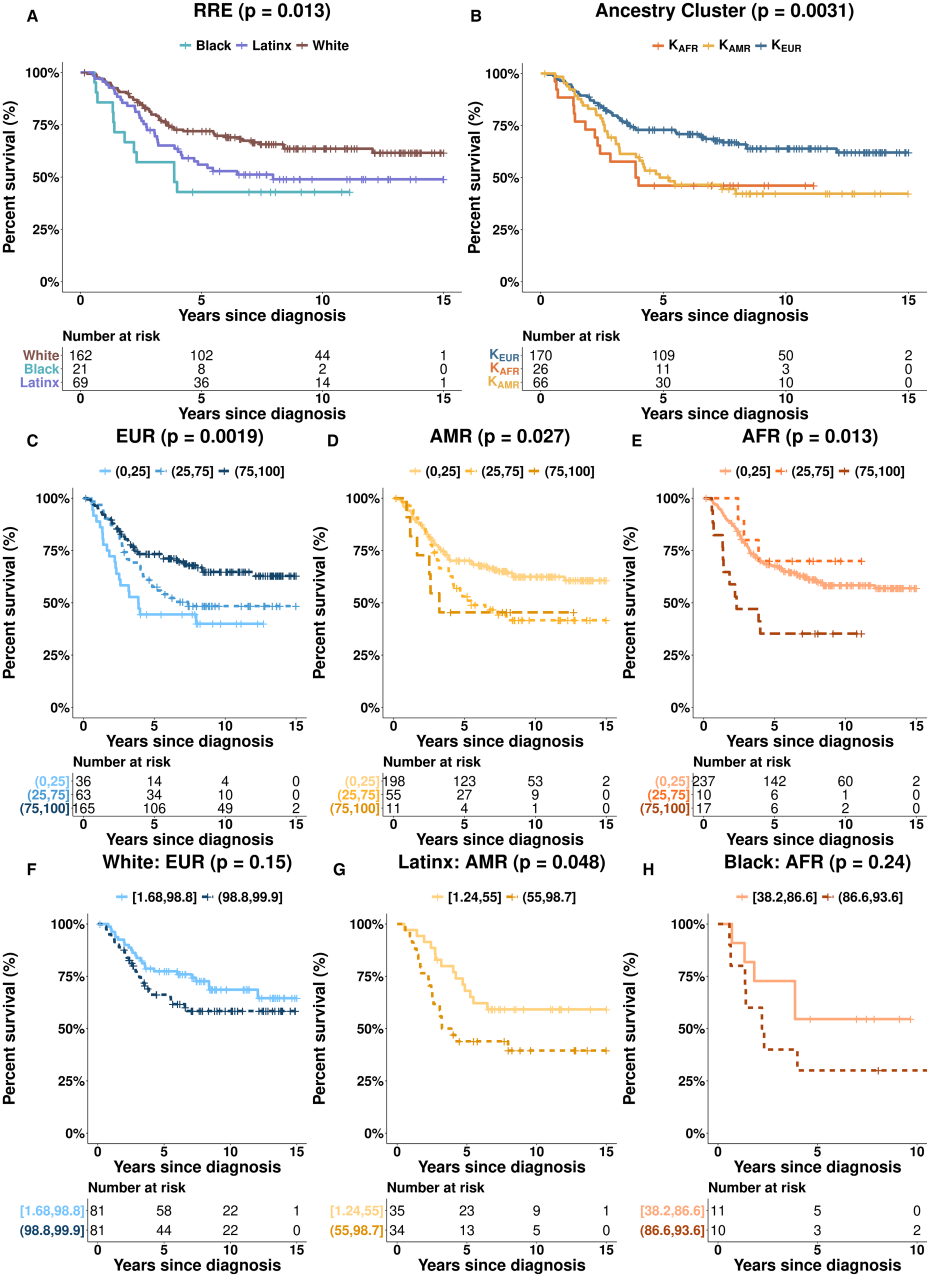
(A) Kaplan Meier survival curves and Log‐Rank *p*‐values for children with B‐ALL by RRE, (B) Kaplan Meier survival curves and Log‐Rank *p*‐values for children with B‐ALL by ancestry cluster, C‐E. Kaplan Meier survival curves and Log‐Rank *p*‐values for children with B‐ALL by genetic ancestry stratified by 0%–25%, 25%–75% and 75%–100% proportions of each ancestry [C. EUR, D. AMR, and E. AFR], F‐H. Kaplan Meier survival curves and Log‐Rank *p*‐values for children with B‐ALL compared by median ancestry proportion within analogous RRE categories [F. EUR proportions among RRE‐White, G. AMR proportions among RRE‐Latinx, and H. AFR proportions among RRE‐Black].

RRE‐Black and RRE‐Latinx children had inferior 15‐year survival compared to RRE‐White children (Log‐Rank *p* = 0.013, Figure 2). Comparing survival across inferred genetic ancestry clusters, we found that K_AFR_ and K_AMR_ children had worse 15‐year survival than K_EUR_ (Log‐Rank *p* = 0.0031, Figure 2B). When creating three categories for ancestry proportion (Figure 2C–E) we found survival significantly improved with increasing EUR ancestry in EUR children (Log‐Rank *p*‐value = 0.0019; Figure 2C). Conversely, for AMR children as AMR ancestry increased, long‐term survival was statistically significant worse (Log‐Rank *p*‐value = 0.027; Figure 2D). For children with AFR ancestry, those with 75%–100% AFR ancestry had worse 15‐year survival than children with <75% AFR ancestry (Log‐Rank *p*‐value = 0.013; Figure 2E). Finally, when predominant ancestry proportions were split at the median within each corresponding RRE group (Figure 2F–H), there were no differences between RRE‐White children with below and above median EUR and no differences between RRE‐Black children with below and above median AFR ancestry (Figure 2F,H). Increasing AMR ancestry in RRE‐Latinx children was associated with statistically worse 15‐year survival (AMR Log‐Rank *p*‐value‐0.048; Figure 2G).

Trends between RRE/ancestry and death were similar in Cox regression analyses when adjusting for relevant clinical factors (Table 2). RRE‐Black and RRE‐Latinx children had an increased risk of death compared to RRE‐White children (RRE‐Black HR: 2.36, 95% CI: 1.20–4.64; RRE‐Latinx HR: 1.59, 95% CI: 0.03–2.46). Trends were similar using K‐means ancestry groups. Considering continuous genetic ancestry proportions, each 10% increase of AFR ancestry and AMR ancestry was associated with increased risks of death (AFR HR: 1.11, 95% CI: 1.02, 1.19; AMR HR: 1.10, 95% CI: 1.02, 1.18). Conversely, 10% increases in EUR ancestry were associated with a decreased risk of death (HR: 0.91, 95% CI: 0.86–0.96). Finally, to explore the role of increasing ancestry in RRE‐defined groups, we found that a 10% increase of AFR genetic ancestry within RRE‐Black children was strongly associated with the risk of death (HR: 5.06; 95% CI: 1.68–15.24).

**TABLE 2:** Hazard ratios and 95% confidence intervals (95% CI) for the association between race/ethnicity or genetic ancestry cluster and proportion and the risk of death adjusting for clinical variables and differentially expressed genes, where noted, in children with B‐ALL, NCI TARGET (2004–2010)

	HR (95% CI)^a^	HR (95% CI)^b^	HR (95% CI)^c^	HR (95% CI)^d^
Reported Race/Ethnicity				
White	Referent	Referent	Referent	Referent
Black	2.36 (1.20, 4.64)	1.84 (0.89, 3.75)	2.12 (0.95, 4.76)	3.35 (1.31, 8.53)
Latinx	1.59 (1.03, 2.46)	1.51 (0.95, 2.39)	1.60 (0.99, 2.57)	1.47 (0.88, 2.45)

K‐Means Ancestry Clusters	
K_EUR_	Referent
K_AFR_	1.91 (1.03, 3.53)
K_AMR_	1.88 (1.22, 2.88)
Each 10% increase in Ancestry Proportion	
EUR	0.91 (0.86, 0.96)
AFR	1.11 (1.02, 1.19)
AMR	1.10 (1.02, 1.18)
RRE‐Stratified each 10% increase in Ancestry Proportion	
White ‐ EUR	0.96 (0.81, 1.14)
Black ‐ AFR	5.07 (1.68, 15.24)
Latinx ‐ AMR	1.16 (0.97, 1.36)

^a^ Model adjusted for sex, age, WBC count, CNS involvement, and cytogenomic subtype.

^b^ Adjusted for sex, age, WBC count, CNS involvement, cytogenomic subtype and log2 expression of DE genes (NUP62, TMEM50B, ATP6AP2) significantly associated with survival after multiple testing adjustment (Benjamini‐Hochberg *p* < 0.05).

^c^ Adjusted for sex, age, WBC count, CNS involvement, cytogenomic subtype and log2 expression of DE genes (NUP62, TMEM50B, ATP6AP2, ZNF586, KANSL1‐AS1, C19orf12) significantly associated with survival without multiple testing adjustment (*p* < 0.05).

^d^ Adjusted for sex, age, WBC count, CNS involvement, molecular subtype and log2 expression of all 22 DE in Table S2.

Next, we explored the role of gene expression within ancestry groups in B‐ALL survival. We identified 21 genes associated with each 10% increase in ancestry proportion (AFR, EUR, AMR) or differentially expressed (DE) by ancestry clusters (K_EUR_ vs K_AFR_, K_EUR_ vs. K_AMR_, K_AMR_ vs. K_AFR_) (Table S2). Of the 21 genes, 10 were associated with clustered and continuous AFR ancestry (*CRYBB2*, *RPTOR*, *PRKCZ*, *TCERG1L*, *LRRC8A*, *HS6ST1*, *TNPO3*, *GTF3C2*, *ZNF586*, and *NUP62*), five genes were associated with EUR (*LINC00667*, *KANSL1‐AS1*, *ATP6AP2*, *TMEM50B*, *ALOX5AP*) and one with AMR (*UTS2*). Three genes were upregulated in K_AFR_ compared to K_EUR_ (*TAFA5*, *UTP4*, *ZNF263*) and two were upregulated in K_AFR_ compared to K_AMR_ (*C19orf12*, *EIFG1*). There were no significant DE genes comparing K_AMR_ to K_EUR_.

**FIGURE 3:**
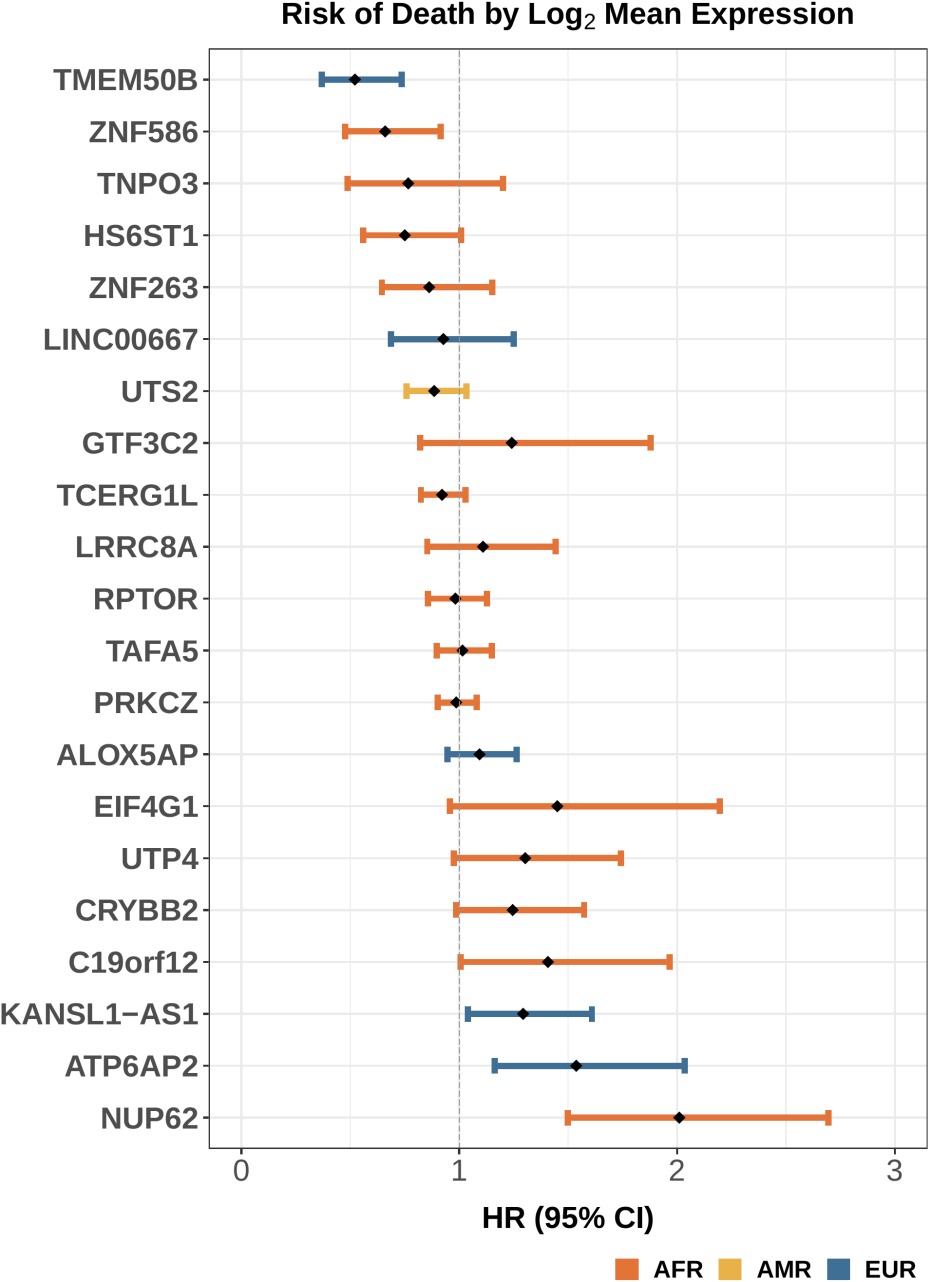
Cox proportional hazards ratios (HR) and 95% confidence intervals (95% CI) for the association between log_2_ mean expression of genes identified as differentially expressed by increasing ancestry proportion or ancestry cluster and death, when adjusting for sex, age, WBC count, CNS involvement, and molecular subtype. Error bars colored by each DE gene's associated ancestry.

Of the 21 genes, four were significantly associated (*p* < 0.05) with an increased risk of death (*NUP62*, *ATP6AP2*, *C19orf12*, *KANSL1‐AS1*) in all children combined and two genes were associated with a decreased risk of death (*TMEM50B* [EUR], *ZNF586* [AFR]; Figure 3, Table S3). Of the genes statistically significantly associated with death, two were associated with AFR ancestry (*C19orf12* and *NUP62)* and two with EUR ancestry (*KANSL1‐AS1, ATP6AP2)*. No AMR‐associated genes were associated with survival.

Finally, we estimated the association between RRE and death, adjusting for ancestry‐associated genes to account for genes of biologic importance in B‐ALL survival (Table 2). Effect estimates were similar in direction and magnitude, though not statistically significant, in models for RRE‐Black and RRE‐Latinx children compared to RRE‐White when adjusting for survival‐associated genes at various statistical thresholds. When adjusting for all 21 genes, the risk of death of RRE‐Black children was over three times that in RRE‐White (HR: 3.35, 95% CI: 1.31–8.53), but the difference between RRE‐Latinx compared to RRE‐White children was null (HR: 1.47, 95% CI: 0.88–2.45). However, these estimates should be interpreted with caution as the sample sizes were small for RRE‐Black children.

## 4 DISCUSSION

In our analysis of TARGET B‐ALL cases, we observed racial/ethnic differences in survival, as reported elsewhere, with RRE‐Latinx and RRE‐Black children faring worse than RRE‐White. We also observed similar differences in outcomes by inferred genetic ancestry. The increased risk of death was most pronounced when considering a 10% increase in ancestry for both AFR and AMR ancestry such that a 50% increase would be associated with 1.69 (AFR HR: 1.11^5) and 1.61 (AMR HR: 1.10^5) times risk of death, respectively. For individuals with 100% AFR and AMR ancestry, the resulting HRs are 2.84 and 2.59, when compared to those with 0% AFR and AMR ancestry, respectively. These estimates are slightly higher than those estimated for RRE‐Black children compared to RRE‐White (HR: 2.36) and much higher than those estimated for RRE‐Latinx children compared to RRE‐White (HR: 1.59) in our study suggesting that genetic ancestry proportion may be a predictor of greater importance for long‐term survival of children with substantial AFR and AMR ancestry. Interestingly, increasing AFR ancestry in RRE‐Black children was associated with 5 times the risk of death, while in other RRE groups there was no association between increasing EUR in RRE‐White and AMR in RRE‐Latinx children and death.

In our analysis, we aimed to identify genes in B‐ALL associated with ancestry that were also associated with survival. We observed 21 genes associated with/DE by ancestry, six of which were also associated with death in analyses with all children (*NUP62*, *ATP6AP2*, *C19orf12*, *KANSL1‐AS1, TMEM50B, and ZNF586)*. In our final analysis of the risk of death by RRE groups adjusting for clinical factors and ancestry‐associated/varying genes, RRE‐Black children had over 3 times the risk of death of RRE‐White suggesting that not accounting for these genes in the analysis blunts observed risk estimates. Overall, our findings suggest that proportions of genetic ancestry are important to consider in B‐ALL survival, particularly among RRE‐Latinx children, where we found a large difference in magnitude between RRE and ancestry‐death estimates. Additionally, understanding the mechanisms by which increasing AFR ancestry increases the risk of death within RRE‐Black children remains crucial. Finally, when accounting for genes associated with ancestry and survival (*n* = 6), the observed association between RRE and death became null in RRE‐Black and RRE‐Latinx children suggesting that consideration of disease biology beyond cytogenomic subtypes may help elucidate mechanisms underlying outcomes. Overall, we observed the expected patterns of genetic ancestry within RRE groups. This observation is consistent with prior research demonstrating heterogeneity in genetic ancestry among RRE groups, despite population‐level correlations between RRE and genetic ancestry.^13^, ^14^, ^20^ In our analyses using RRE/K‐means genetic ancestry to examine B‐ALL survival, RRE‐White and K_EUR_ children had lower risks of death than other RRE/ancestry cluster groups. These results are consistent with prior literature demonstrating poorer outcomes among Black and Hispanic/Latinx compared to White children.^23^ When using genetic ancestry as a continuous measure, we observed statistically significant increased risks of death for non‐EUR groups and a statistically significant inverse association with death for increasing EUR ancestry proportion even after stratifying by RRE, which we used as a proxy measure for socioeconomic status and other factors that differ by socially‐defined race/ethnicity. The findings for genetic ancestry‐defined AFR individuals versus EUR are similar to those recently reported for a type of B‐cell lymphoma suggesting consideration of genetic ancestry may be important across hematologic malignancies.^24^

We found 21 genes associated with ancestry in children with B‐ALL. Of note, *CRYBB2* is DE by RRE in diseased and normal tissue and is associated with poor prognosis in breast cancer.^25^, ^26^ *CRYBB2* in germline analyses is associated with prostate cancer in Black men.^27^ In our analyses *CRYBB2* was the top gene associated with AFR ancestry in children with B‐ALL suggesting that it may play a more global, ancestry‐dependent role in carcinogenesis in children and adults. *NUP62*, a nucleoporin gene family member, was associated with AFR ancestry and was the gene mostly strongly associated with death in our study. *NUP62* has been associated with autoimmune conditions and ovarian cancer.^28^ The role for *NUP62* in B‐ALL risk and outcome remains to be explored. Confirmatory and mechanistic studies are needed to better understand the role of the genes we identified in B‐ALL.

Our study is not without limitations. It is possible that subjects with missing subtype information had molecular subtypes aside from the 9 subtypes considered in our inference; however, TARGET had no documentation of such cases and subtype clustering effects (Figure S1) were too weak to clearly identify subtypes beyond the included 9 from the TARGET data. Although TARGET data had relatively complete RRE, it is unclear how it was collected (self‐identified, physician‐reported, etc.). Our analyses of ancestry and death assumed that residual disparities in B‐ALL survival were captured in RRE categories as TARGET lacks socioeconomic data. Due to the absence of treatment‐related mortality data and sample size constraints, we were unable to distinguish the role of treatment‐related toxicity from the relationship between ancestry and overall survival. Genetic ancestry classification cutoffs and methods vary by study, hindering comparisons across studies. When we simplify ancestry^29^ classification results into discrete categories (i.e. using K‐means clusters), we may miss importance nuances of the biologic genetic ancestry. As TARGET was a multisite study with limited institutional information, we cannot account for institution‐specific treatment protocols which is a meaningful limitation when studying long‐term mortality. However, by accounting for data collection phases and baseline risk measures, we aimed to minimize biasing effects due to institutional protocols. Additionally, TARGET B‐ALL cases were all high‐risk and as such the observed survival differences may only apply to the population of children with high‐risk BALL and not the full spectrum of disease. The availability of microarray rather than RNAseq data limited the number of DE genes identified, so our findings should be validated and expanded upon in RRE‐diverse datasets with RNAseq, genotype, and survival data in children with B‐ALL.

To conclude, we observed poor survival in RRE‐Black, RRE‐Latinx, predominantly African ancestry, and predominantly Amerindian ancestry children with B‐ALL when adjusting for clinical risk factors. These findings were most pronounced when genetic ancestry was considered as a continuous variable suggesting increasing proportions of non‐EUR ancestry are associated with higher risk of death among children with B‐ALL independent of known prognostic factors such as subtype or ALL risk group, which may impact treatment development and delivery. We found 21 ancestry associated genes that when accounted for in Cox regression models increased the magnitude of the association between Black RRE and death while the findings were non‐significant for RRE‐Latinx children. However, after adjustment for only the six DE genes associated with survival in all children under study, there was no evidence of different survival between RRE‐Black or RRE‐Latinx children compared to RRE‐White. Collectively, our work suggests we must consider genetic ancestry and associated genes when developing risk prediction models, new therapies, and personalized medicine approaches rather than continuing to rely on RRE. Further, our findings highlight the additional information that can be gleaned from studies of racial/ethnic disparities when additionally considering genetic ancestry alongside RRE information in B‐ALL long‐term survival in children.

## AUTHOR CONTRIBUTIONS

**Freddy Barragan:** Conceptualization (equal); data curation (lead); formal analysis (lead); investigation (lead); methodology (equal); software (lead); visualization (lead); writing – original draft (equal); writing – review and editing (lead). **Lauren J Mills:** Conceptualization (equal); formal analysis (supporting); investigation (supporting); methodology (equal); project administration (equal); software (supporting); supervision (equal); writing – original draft (equal). **Andrew Ronald Raduski:** Data curation (equal); formal analysis (supporting); investigation (supporting); project administration (supporting); software (supporting); supervision (equal); writing – original draft (equal). **Erin Marcotte:** Conceptualization (equal); investigation (supporting); supervision (equal); writing – original draft (equal). **Kelsey Erin Grinde:** Formal analysis (supporting); funding acquisition (supporting); investigation (equal); methodology (lead); software (equal); supervision (equal); writing – original draft (equal). **Logan G. Spector:** Conceptualization (lead); funding acquisition (lead); investigation (equal); project administration (lead); resources (equal); supervision (equal); writing – original draft (equal). **Lindsay A Williams:** Conceptualization (lead); funding acquisition (lead); investigation (lead); methodology (lead); project administration (lead); supervision (equal); writing – original draft (lead); writing – review and editing (equal).

## FUNDING INFORMATION

This work was supported by the National Cancer Institute of the National Institutes of Health under Award Number 1R01CA239701‐01A1S1 (PI: LGS; Trainee: FB). This work is also supported by the Children's Cancer Research Fund and the Department of Defense (W81XWH‐21‐1‐0397; LAW).

## CONFLICT OF INTEREST

The authors have no conflicts of interest to declare.

## Supporting information


Table S1
Table S2
Table S3
Figure S1
Figure S2
Figure S3

## Data Availability

The results herein are based on data generated by the TARGET initiative (https://ocg.cancer.gov/programs/target), phs000218 (https://portal.gdc.cancer.gov/projects)
