# Supplementary material for: Genetic ancestry, differential gene expression, and survival in pediatric B‐cell acute lymphoblastic leukemia
Source: Cancer Med. 2022 Sep 20;12(4):4761–72. doi: 10.1002/cam4.5266 (PMC9972134; doi:10.1002/cam4.5266)

**Supplementary Information**

**Supplemental Table 1. Hazards ratios (HR) and 95 % confidence intervals (95% CI) for the association between RRE, ancestry cluster, each 10% increase in ancestry proportion, and each 10% increase in ancestry proportion within RRE categories in children with B-ALL at 5-,10-, and 15- year survival, NCI TARGET (2004-2010)**

|  | **5-Year HR (95% CI)** | **10-Year (95% CI)** | **15-Year (95% CI)** |
| --- | --- | --- | --- |
| **RRE** | | | |
| **White** | Referent | Referent | Referent |
| **Black** | 2.49 (1.27, 4.92) | 2.13 (1.10, 4.13) | 2.14 (1.10, 4.13) |
| **Latinx** | 1.84 (1.17, 2.90) | 1.73 (1.14, 2.62) | 1.71 (1.13, 2.59) |
| **Ancestry Cluster** | | | |
| **K_EUR_** | Referent | Referent | Referent |
| **K_AFR_** | 2.39 (1.28, 4.47) | 2.02 (1.10, 3.73) | 2.03 (1.10, 3.74) |
| **K_AMR_** | 2.01 (1.27, 3.19) | 1.89 (1.23, 2.90) | 1.88 (1.23, 2.88) |
| **Ancestry Proportion** | | | |
| **EUR** | 0.89 (0.83, 0.94) | 0.9 (0.86, 0.95) | 0.9 (0.86, 0.96) |
| **AFR** | 1.13 (1.04, 1.22) | 1.1 (1.02, 1.20) | 1.1 (1.02, 1.20) |
| **AMR** | 1.12 (1.05, 1.20) | 1.11 (1.04, 1.18) | 1.11 (1.04, 1.18) |
| **RRE-Stratified Ancestry Proportion** | | | |
| **White - EUR** | 0.97 (0.83, 1.17) | 0.97 (0.82, 1.15) | 0.97 (0.82, 1.15) |
| **Black - AFR** | 4.15 (1.7, 9.89) | 4.15 (1.74, 9.89) | 4.15 (1.74, 9.89) |
| **Latinx - AMR** | 1.2 (1.02, 1.43) | 1.16 (0.99, 1.36) | 1.16 (0.99, 1.36) |

**Supplemental Table 2. Statistically significantly differentially expressed genes by ancestry proportion (each 10% increase) or between ancestry clusters in children with B-ALL, NCI TARGET (2004-2010)**

| **AFR Ancestry^1^** | | | | | | | | | | |
| --- | --- | --- | --- | --- | --- | --- | --- | --- | --- | --- |
| **Model** | **Symbol** | **logFC** | **AveExpr** | **t** | **p-value** | **adj.p** | **b** | **probeID** | **chr** |  |
| Proportion | CRYBB2 | 51.40155 | 207.5005 | 10.30659 | 0 | 0 | 17.30102 | ENSG00000244752 | 22 |  |
| Proportion | RPTOR | 79.98943 | 340.2829 | 7.494135 | 0 | 0 | 8.154633 | ENSG00000141564 | 17 |  |
| Proportion | PRKCZ | 500.6474 | 2206.633 | 5.510091 | 1E-07 | 0.000424 | 2.575672 | ENSG00000067606 | 1 |  |
| Proportion | TCERG1L | 178.1013 | 560.7525 | 5.507953 | 1E-07 | 0.000424 | 2.567911 | ENSG00000176769 | 10 |  |
| Proportion | LRRC8A | 153.3584 | 1821.487 | 5.34004 | 2E-07 | 0.000785 | 2.153497 | ENSG00000136802 | 9 |  |
| Proportion | HS6ST1 | 34.25988 | 404.8817 | 5.047949 | 8E-07 | 0.002724 | 1.443105 | ENSG00000136720 | 2 |  |
| Proportion | TNPO3 | 40.03358 | 1033.664 | 4.96323 | 1E-06 | 0.003492 | 1.242597 | ENSG00000064419 | 7 |  |
| Proportion | GTF3C2 | 44.33685 | 972.3868 | 4.895801 | 2E-06 | 0.003685 | 1.084837 | ENSG00000115207 | 2 |  |
| Proportion | ZNF586 | 36.5724 | 772.8507 | 4.873844 | 2E-06 | 0.003685 | 1.033818 | ENSG00000083828 | 19 |  |
| Proportion | NUP62 | 32.78092 | 498.0101 | 4.860734 | 2E-06 | 0.003685 | 1.003439 | ENSG00000213024 | 19 |  |
| AFR v EUR | TAFA5 | 373.7335 | 250.2615 | 4.731433 | 4.3E-06 | 0.0143367 | -4.408917 | ENSG00000219438 | 22 |  |
| AFR v EUR | UTP4 | 930.1858 | 2018.6637 | 4.573423 | 8.5E-06 | 0.0212664 | -4.420333 | ENSG00000141076 | 16 |  |
| AFR v EUR | ZNF263 | 656.8899 | 1470.7853 | 4.379864 | 1.9E-05 | 0.0386648 | -4.434263 | ENSG00000006194 | 16 |  |
| AFR v AMR | C19orf12 | 301.3657 | 499.395 | 5.20783 | 1.2E-06 | 0.0241472 | -4.416126 | ENSG00000131943 | 19 |  |
| AFR v AMR | EIF4G1 | 449.6576 | 1154.194 | 4.923554 | 4E-06 | 0.025641 | -4.43186 | ENSG00000114867 | 3 |  |
| **AMR Ancestry^1^** | | | | | | | | | | |
| Proportion | UTS2 | 20.28144 | 133.8495 | 5.061587 | 8.00E-07 | 0.015311 | 2.53462 | ENSG00000049247 | 1 |  |
| **EUR Ancestry^1^** | | | | | | | | | | |
| Proportion | LINC00667 | 13.85719 | 339.6403 | 5.128454 | 6.00E-07 | 0.011096 | 3.965468 | ENSG00000263753 | 18 |  |
| Proportion | KANSL1-AS1 | 35.62976 | 473.2425 | 4.898981 | 1.70E-06 | 0.01192 | 3.197637 | ENSG00000214401 | 17 |  |
| Proportion | ATP6AP2 | 112.4764 | 2332.085 | 4.882104 | 1.80E-06 | 0.01192 | 3.144092 | ENSG00000182220 | X |  |
| Proportion | TMEM50B | 24.88769 | 736.8166 | 4.58944 | 6.80E-06 | 0.028938 | 2.210311 | ENSG00000142188 | 21 |  |
| Proportion | ALOX5AP | 222.1664 | 2676.316 | 4.574862 | 7.30E-06 | 0.028938 | 2.164495 | ENSG00000132965 | 13 |  |

^1^DE models adjusted for phase and molecular subtype. adj.p-value = Benjamini-Hochberg adjusted p-values.

**Supplemental Table 3. Hazards ratios (HR) and 95 % confidence intervals (95% CI) for the association between differentially expressed genes log2 mean and death in children with B-ALL (bolded estimates exclude the null value of 1), NCI TARGET (2004-2010)**

| **Gene Symbol** | **HR^1^** | **95% CI** | **p-value** | **BH p-value** |
| --- | --- | --- | --- | --- |
| **NUP62** | **2.01** | **(1.49, 2.69)** | **0.0000032** | **0.0000667** |
| **TMEM50B** | **0.52** | **(0.36, 0.73)** | **0.0002175** | **0.0022837** |
| **ATP6AP2** | **1.53** | **(1.16, 2.03)** | **0.0026097** | **0.0182677** |
| **ZNF586** | **0.66** | **(0.47, 0.91)** | **0.0123935** | **0.0650657** |
| **KANSL1-AS1** | **1.29** | **(1.03, 1.61)** | **0.0214366** | **0.0900338** |
| **C19orf12** | **1.40** | **(1.01, 1.96)** | **0.0455561** | **0.1594462** |
| HS6ST1 | 0.75 | (0.56, 1.01) | 0.0563199 | 0.1674709 |
| CRYBB2 | 1.24 | (0.98, 1.57) | 0.0662383 | 0.1674709 |
| UTP4 | 1.30 | (0.97, 1.74) | 0.0742363 | 0.1674709 |
| EIF4G1 | 1.45 | (0.95, 2.19) | 0.0797480 | 0.1674709 |
| UTS2 | 0.88 | (0.75, 1.03) | 0.1229724 | 0.2347655 |
| TCERG1L | 0.92 | (0.82, 1.03) | 0.1418739 | 0.2482794 |
| ALOX5AP | 1.09 | (0.94, 1.26) | 0.2305318 | 0.3660019 |
| TNPO3 | 0.76 | (0.48, 1.20) | 0.2440013 | 0.3660019 |
| GTF3C2 | 1.24 | (0.82, 1.88) | 0.3071387 | 0.4131853 |
| ZNF263 | 0.86 | (0.64, 1.15) | 0.3148078 | 0.4131853 |
| LRRC8A | 1.11 | (0.85, 1.44) | 0.4400933 | 0.5436447 |
| LINC00667 | 0.93 | (0.68, 1.25) | 0.6182293 | 0.7212675 |
| PRKCZ | 0.99 | (0.90, 1.08) | 0.7622092 | 0.8185078 |
| RPTOR | 0.98 | (0.85, 1.13) | 0.7979713 | 0.8185078 |
| TAFA5 | 1.02 | (0.89, 1.15) | 0.8185078 | 0.8185078 |

^1^Models adjusted for RRE, sex, age, WBC count, CNS involvement, and cytogenetic subtype.

**Supplemental Figure 1A. Principal component analysis of gene expression data along PC1 & PC2 for classification of cytogenomic subtypes in children with B-ALL. Color indicates a known subtype of the patient, while smaller grey points indicate an unknown subtype, which was inferred; 1B. Random forest classification boundaries of subtype classification after inference analysis along PC1 & PC2. Color indicates the subtype classification our RF classifier would assign at these PC1 & PC2 values, NCI TARGET (2004-2010)**

**
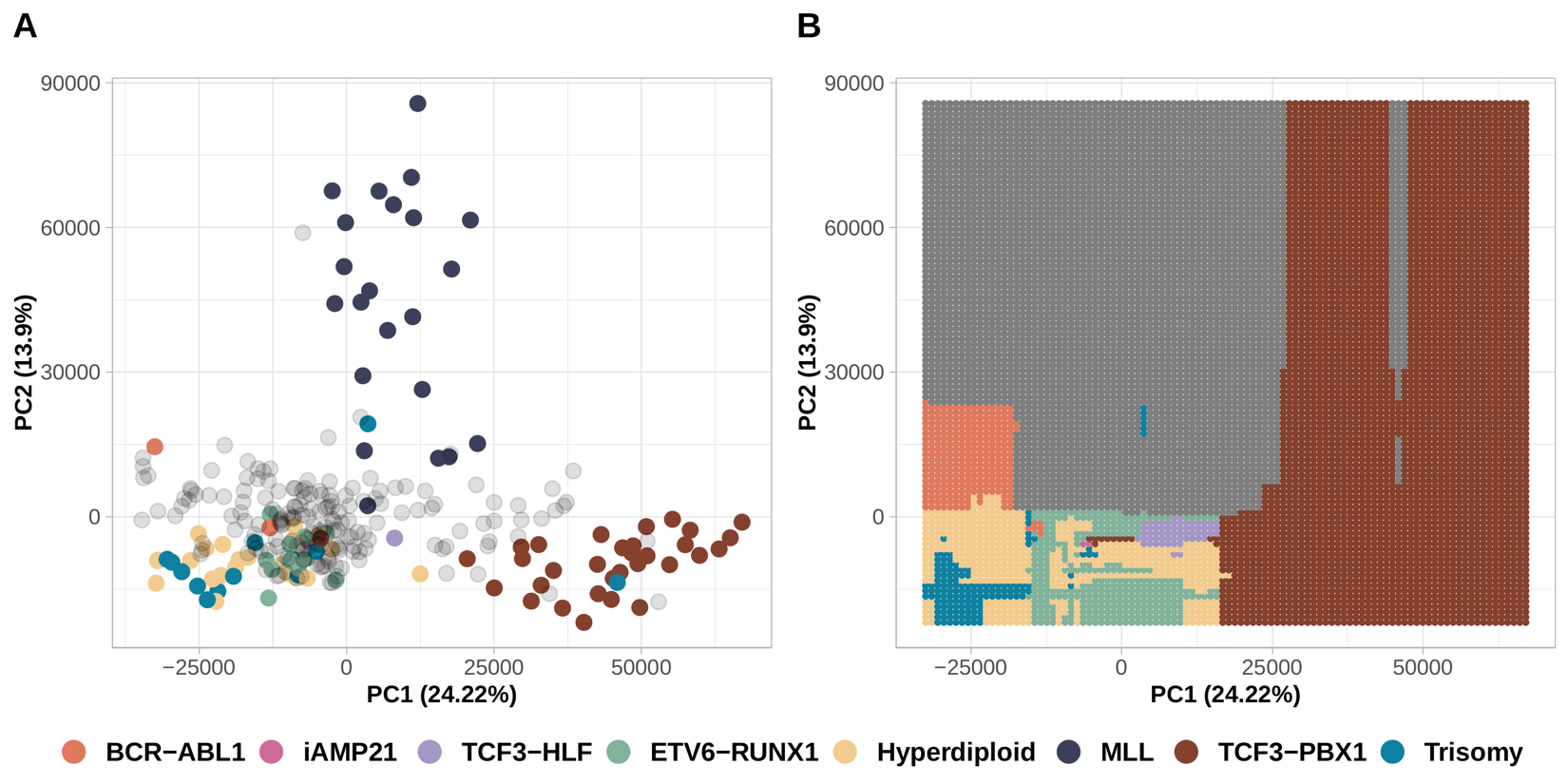
**

**Supplemental Figure 2. Genetic ancestry proportions of B-ALL cases colored by K-means cluster assignment (A) and RRE (B), NCI TARGET, (2004-2010)**


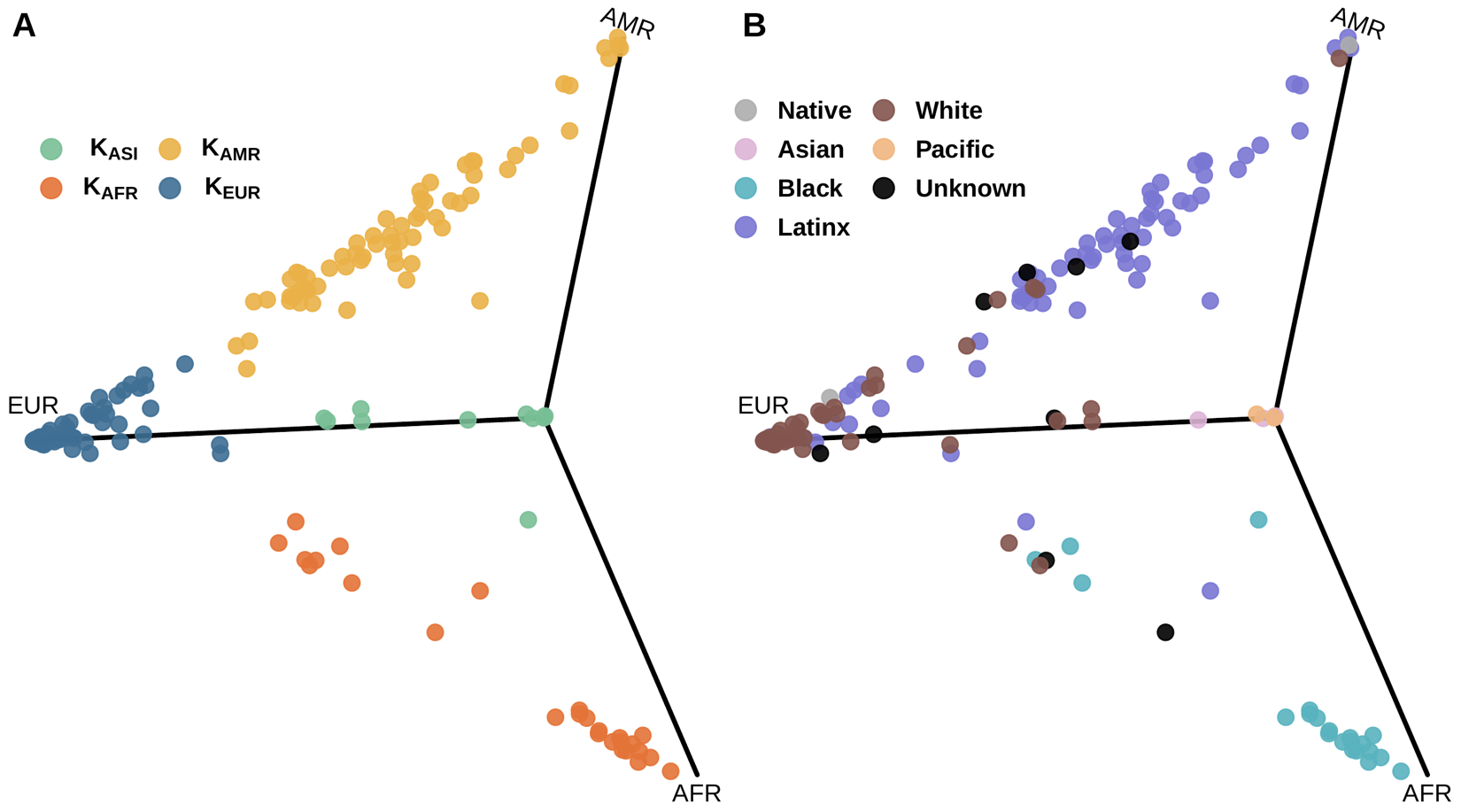


**Supplemental Figure 3. Cross classification of RRE within K-means cluster ancestry categories, NCI TARGET, (2004-2010)**


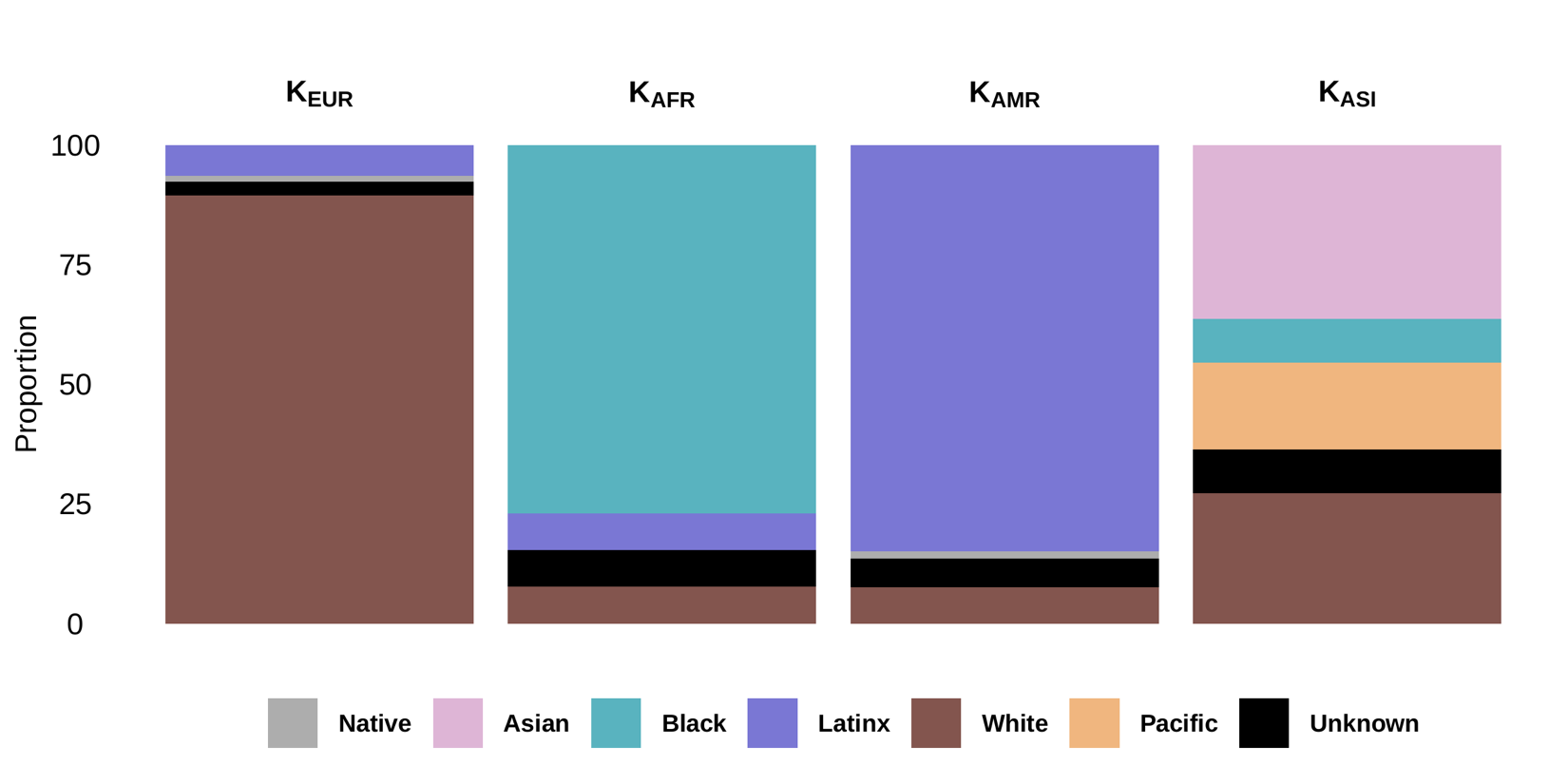

Supplement: Supplementary file 1 — Table S1 Table S2 Table S3 Figure S1 Figure S2 Figure S3 [file CAM4-12-4761-s001.docx]
